# Smyd2 conformational changes in response to p53 binding: role of the C‐terminal domain

**DOI:** 10.1002/1878-0261.12502

**Published:** 2019-05-21

**Authors:** Balasubramanian Chandramouli, Gerry Melino, Giovanni Chillemi

**Affiliations:** ^1^ Scuola Normale Superiore Pisa Italy; ^2^ Department of Experimental Medicine TOR, University of Rome ‘Tor Vergata’ Italy; ^3^ Medical Research Council, Toxicology Unit Department of Pathology, Cambridge University Cambridge UK; ^4^ Department for Innovation in Biological, Agro‐Food and Forest Systems (DIBAF) University of Tuscia Viterbo Italy; ^5^ National Council of Research CNR Institute of Biomembranes, Bioenergetics and Molecular Biotechnologies Bari Italy; ^6^ Present address: Compunet Istituto Italiano di Tecnologia (IIT) Genova Italy

**Keywords:** AdoMet, C‐terminal domain, lysine methylation, molecular dynamics, p53, Smyd2

## Abstract

Smyd2 lysine methyltransferase regulates monomethylation of histone and nonhistone lysine residues using S‐adenosylmethionine cofactor as the methyl donor. The nonhistone interactors include several tumorigenic targets, including p53. Understanding this interaction would allow the structural principles that underpin Smyd2‐mediated p53 methylation to be elucidated. Here, we performed μ‐second molecular dynamics (MD) simulations on binary Smyd2‐cofactor and ternary Smyd2‐cofactor‐p53 peptide complexes. We considered both unmethylated and monomethylated p53 peptides (at Lys370 and Lys372). The results indicate that (a) the degree of conformational freedom of the C‐terminal domain of Smyd2 is restricted by the presence of the p53 peptide substrate, (b) the Smyd2 C‐terminal domain shows distinct dynamic properties when interacting with unmethylated and methylated p53 peptides, and (c) Lys372 methylation confines the p53 peptide conformation, with detectable influence on Lys370 accessibility to the cofactor. These MD results are therefore of relevance for studying the biology of p53 in cancer progression.

**Abbreviations**
MD: molecular dynamics
MYND: myeloid‐nervy‐DEAF1
PCA: principal component analysis
PDB: protein data bank
RMSF: root‐mean‐square fluctuation
SET: suppressor of variegation, enhancer of zeste, trithorax

## 1 Introduction

Smyd2 is a member of five lysine methyltransferases constituted by a suppressor of variegation, enhancer of zeste, trithorax (SET) domain (InterPro ID IPR001214), intercalated by a myeloid‐nervy‐DEAF1 (MYND) zinc binding domain (InterPro ID IPR002893). Smyds catalyze the monomethylation of histone and nonhistone lysine residues, using the S‐adenosylmethionine methyl donor cofactor (AdoMet/SAM) and releasing the product S‐adenosylhomocysteine (AdoHcy/SAH) (Spellmon *et al*., 2015).

Smyd2 targets H3 (Lys4, Lys36), but its peculiarity among the Smyds lies in the vast broad spectrum of nonhistone targets that comprehend around twenty proteins, such as p53 (Lys370), retinoblastoma (Lys860), estrogen receptor ERα (Lys266), PARP1 (Lys528), HSP90 (Lys531, Lys574), and, recently, BMPR2 (Brown *et al*., 2006; Foreman *et al*., 2011; Gao *et al*., 2017; Gottlieb *et al*., 2002; Hamamoto *et al*., 2004; Van Aller *et al*., 2012). Recent proteomics studies have identified several additional potential Smyd2 substrates (Ahmed *et al*., 2016; Olsen *et al*., 2016).

Another methyltransferase, Set9, also targets p53 (at Lys372) (Chuikov *et al*., 2004). This may be of particular interest in DNA damage response (Nemajerova *et al*., 2018; Parrales *et al*., 2018), cell cycle arrest (Engeland, 2018; Wu and Prives, 2018), tumor suppression (Charni *et al*., 2018; Kaiser and Attardi, 2018) and in particular in cancer progression (Aubrey *et al*., 2018; Baugh *et al*., 2018; Kim and Lozano, 2018), when considering the pivotal role of p53 in these molecular mechanisms (Furth and Aylon, 2017; Furth *et al*., 2018; Sullivan *et al*., 2018). Consequently, the interaction between p53 and Smyd2 could be of high relevance. Methylation of Lys370 p53 by Smyd2 reduces its binding efficiency to promoter genes, thereby repressing p53 transcriptional activity (Huang *et al*., 2006). The opposite functional effect is observed when Set9 methylates Lys372, with an increase of stability and activity of p53 (Marouco *et al*., 2013). A fine tuning of p53 transcriptional activity, therefore, is obtained via alternative methylation by Smyd2 on Lys370 and Set9 on Lys372.

Different *in vivo* studies have demonstrated the role played by Smyd2 in cancer initiation and progression (Bagislar *et al*., 2016; Reynoird *et al*., 2016). In line, downregulation of Smyd2 by small interfering RNA in cells promotes p53‐mediated apoptosis, while its overexpression in esophageal squamous cell carcinoma is inversely correlated with patients survival rate (Komatsu *et al*., 2009). Smyd2 is also highly expressed in pediatric acute lymphoblastic leukemia, and higher expression level is correlated with bad prognosis (Sakamoto *et al*., 2014). The important role played by Smyd2 in cancer biology has spurred the development of cell‐active inhibitors (Ferguson *et al*., 2011; Nguyen *et al*., 2015; Sweis *et al*., 2015). Recent evidences, however, show no impact of Smyd2–3 inhibition on cancer cell proliferation *in vitro* (Thomenius *et al*., 2018). Smyd2 and 3, therefore, could play a role in other steps of oncogenesis such as an early initiation step of oncogenesis, or in cell growth relevant only *in vivo*, like the regulation of the tumor microenvironment, angiogenesis, or immune evasion (Mazur *et al*., 2014; Thomenius *et al*., 2018). Further studies on the functional mechanism of Smyds are clearly needed.

**Figure 1:**
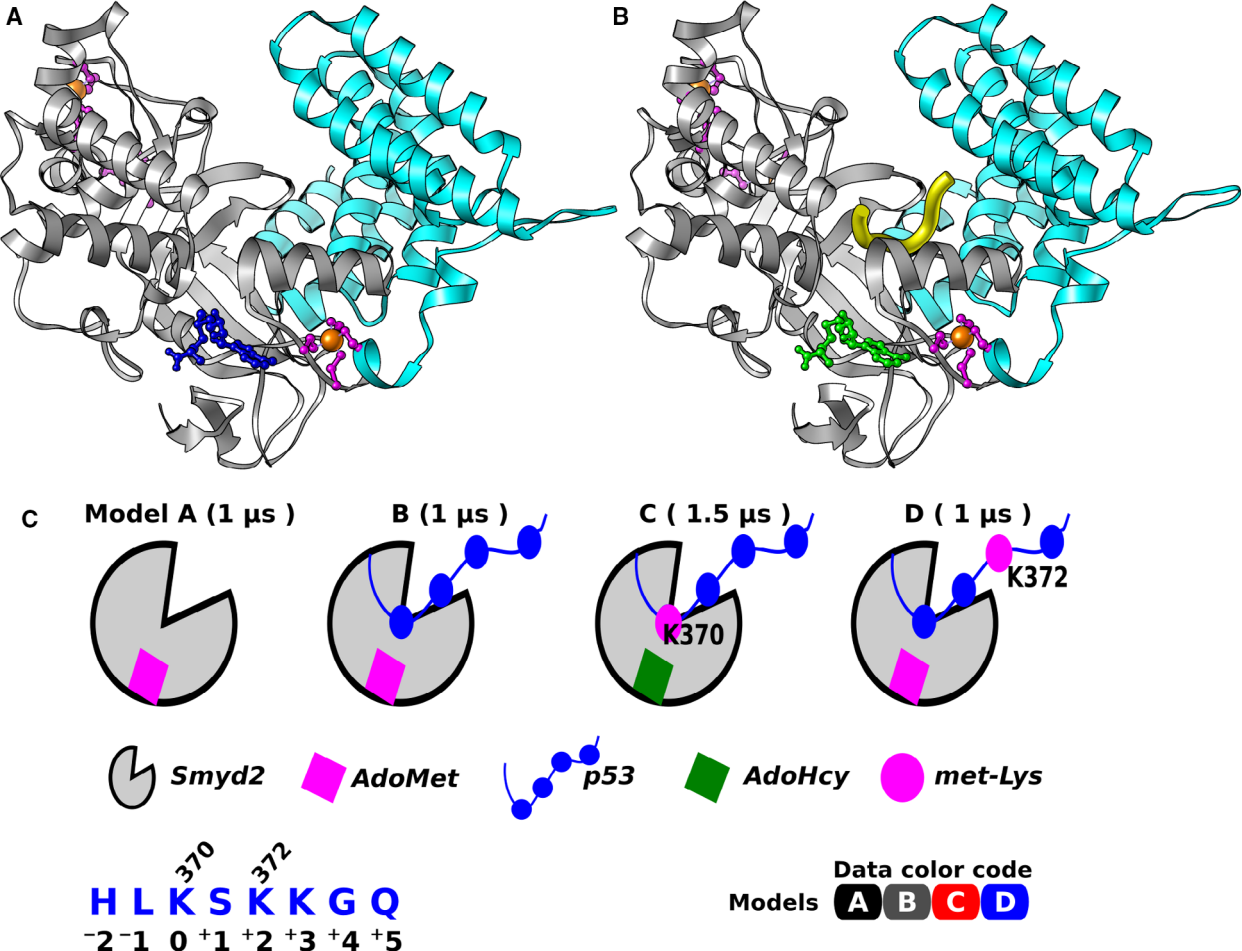
Smyd2 structure and simulated complex models. X‐ray structures of binary Smyd2‐AdoMet (A) and ternary Smyd2‐AdoHcy‐p53 (B) complex. Smyd2 N‐ and C‐terminal domains are shown as ribbons in gray and cyan. AdoMet and AdoHcy are represented in blue and green, respectively. Zinc ions are shown as orange spheres with coordinated protein residues in magenta. The p53 peptide is shown as yellow tube. (C) Schematic view of the simulated models. The sequence numbering of the p53 peptide is given for clarity. The Data color code adopted in all the following graphs is also shown.

From a structural point of view, the N‐ and C‐terminal domains of Smyd2 form a clamp in which the substrate is inserted in a position in which it can receive the methyl group from AdoMet (Fig. 1A,B). Recently, we carried out a molecular dynamical study of Smyd2, highlighting its differential substrate crevice characteristics as compared to Smyd3 (Chandramouli and Chillemi, 2016; Chandramouli *et al*., 2016). From these studies, a critical role of the Smyds C‐terminal domain emerges that orchestrates the catalytic cycle.

Previously, we have also performed dynamic studies on p53 in monomeric (Chillemi *et al*., 2013) and tetrameric (Chillemi *et al*., 2017; D'Abramo *et al*., 2016) conformation, showing a relevant induced‐fit interaction of the p53 C‐terminal domain with the DNA‐binding domain. Here, we have performed μ‐second molecular dynamics (MD) simulations to further understand the underlying molecular events regulating the Smyd2 catalytic cycle.

## 2 Materials and methods

### 2.1 Model generation and simulation protocol

Atomic coordinates for binary Smyd2‐AdoMet (PDB ID: 3TG4) and ternary Smyd2‐AdoHcy‐p53 (PDB ID: 3TG5) complexes were extracted from better resolved X‐ray crystallographic structures from PDB (Wang *et al*., 2011). Histidine protonation states were assigned based on the consensus predictions by H^++^ (v3.1), Protoss and Propka programs (Anandakrishnan *et al*., 2012; Bietz *et al*., 2014; Li *et al*., 2005). All simulations were performed with amber package (v.14) treating the protein with ff14SB forcefield (Hornak *et al*., 2006). Parameters for AdoMet and AdoHcy were adapted from Amber ff10 forcefield and a previous study (Hornak *et al*., 2006; Markham *et al*., 2002; Wang *et al*., 2000). The partial charges were obtained after a geometry optimization at HF/6‐31G* level of theory using Gaussian (Gaussian 09; Gaussian Inc., Wallingford, CT, USA), followed by fitting the electrostatic potential with the restrained electrostatic potential procedure using RED tools (Bayly *et al*., 1993; Vanquelef *et al*., 2011). Dihedral restraints were applied during optimization to retain enzyme‐bound conformations of AdoMet and AdoHcy. Zinc ions and the associated protein residues coordinated to the ion were treated with ZAFF parameters (Li *et al*., 2013; Peters *et al*., 2010). The starting structures were placed in a cubic box of TIP3P water model that extended up to ~ 15 Å from solute surface (Jorgensen *et al*., 1983). Additional counterions were added to achieve charge neutrality. All systems were subjected to elaborate equilibration as detailed in our previous reports (Chandramouli and Chillemi, 2016; Chandramouli *et al*., 2016). Production simulations were performed in NVT ensemble for 1 μs for each system, and snapshots in the trajectory were saved at intervals of 10 ps. The simulation conditions included periodic boundary conditions, constraining covalent heavy atom–hydrogen bonds via SHAKE (Ryckaert *et al*., 1977), 2 fs time step for numerical integration, PME method for calculating long‐range electrostatic interactions (Darden *et al*., 1993), 12 Å cutoff for short‐range nonbonded interactions, temperature regulation with Langevin coupling using a collision frequency of 2.0 ps^−1^ (Izaguirre *et al*., 2001).

### 2.2 MD Trajectories analysis

For all root‐mean‐square (RMS) fitting, the respective X‐ray structures were used as the reference. Unless stated otherwise, reference to backbone means C_α_, C, N atoms. Dynamic cross‐correlation matrix was computed considering the backbone coordinates as follows,

$$C_{\mathrm{ij}}=\frac{\left\langle \Delta r_{i}\Delta r_{j}\right\rangle }{\sqrt{\left\langle \Delta r_{i}^{2}\right\rangle }\sqrt{\left\langle \Delta r_{j}^{2}\right\rangle }}$$

where Δ*r*_*i*_ is the displacement of *i*th atom from the mean position and < > represents the ensemble average over the analyzed portion of trajectory. Interaction energies and atom distances were calculated on the last 900 ns portion of the trajectories considering a snapshot every 100 ps (for a total of 9000 analyzed conformations). Principal component analysis (PCA) was performed by diagonalizing the covariance matrix obtained from atomic fluctuations after removing rotations and translations by fitting the snapshots onto the X‐ray structure (Amadei *et al*., 1993). To this purpose, a concatenated artificial trajectory was generated with snapshots from respective simulations obtained at 100 ps intervals over the last 900 ns. This permitted to project the frames yielded from the independent simulations on a common plane. Geometric angles (Chandramouli and Chillemi, 2016 for definitions) were calculated after fitting the trajectories over the N‐terminal domain of the X‐ray structures. All above analyses were performed using ambertools (v1.5), gromacs utilities (v.5.0.7) or codes in‐house written using MDAnalysis (Michaud‐Agrawal *et al*., 2011; Roe and Cheatham, 2013; Van Der Spoel *et al*., 2005). Graphical plots and figures were generated with Matplotlib library and UCSF Chimera (Hunter, 2007; Pettersen *et al*., 2004). Clustering was performed using hierarchical density‐based method as implemented in hdbscan library (Campello *et al*., 2013; Campello *et al*., 2015; McInnes *et al*., 2017).

## 3 Results and Discussion

In order to improve our understanding of the Smyd2 catalytic cycle, here we carried out μ‐second MD simulations of the following systems (see Fig. 1): Smyd2 in complex with AdoMet cofactor (Model‐A); Smyd2‐AdoMet‐p53 peptide (unmethylated) complex (Model‐B); Smyd2‐AdoHcy‐p53 peptide (methylated at Lys370) complex (Model‐C); and Smyd2‐AdoMet in complex with the p53 peptide methylated at Lys372 (Model‐D).

In the following, we report structural elements that describe the conformational rearrangements and flexibility of Smyd2 and p53 peptides; the global motion of Smyd2 protein associated in its binary and ternary states, and finally, we highlight the dynamical characteristics of unmethylated and methylated p53 peptides as they bound to Smyd2 (see Fig. S1). The following color code is adopted in the figures: Model‐A (black), Model‐B (gray), Model‐C (red), and Model‐D (blue), as reported also in Fig. 1C.

### 3.1 Peptide substrates restrict the motion of Smyd2 C‐terminal domain

The structural rearrangements during the simulation with respect to the starting X‐ray configuration were monitored via estimating the backbone RMSD for Smyd2 and p53 peptides (Fig. S2). The time evolution of RMSD values shows a higher deviation in the binary system (Model‐A) compared to the ternary systems (Fig. S2A), and the RMSD values are uniformly distributed around a higher mean of 3.2 (± 0.4). This indicates a larger structural relaxation in the absence of the substrate peptide. Among the ternary systems, RMSD values of Model D (p53 peptide with methylated Lys372) show a remarkable stability, while the two models B and C (unmethylated lysines and methylated at Lys370, respectively) fluctuate during the simulations up to 3 Å. The mean RMSD for models B–D are 2.0 (± 0.3), 1.9 (± 0.5), and 1.4 (± 0.2) Å, respectively. First, there results already show the great influence played by the bound p53 peptide on the overall dynamics of Smyd2, including the capability to discriminate between the two methylated lysines.

Further, the distribution of RMSD values calculated separately for Smyd2′s N‐ and C‐terminal domains (hereinafter, NTD and CTD) shows a high overlap for the NTD across the all simulated models (Fig. S2B). For the CTD, interestingly, the RMSD distribution in the binary case is shifted toward higher values with respect to the ternary ones. Overall, these results imply that the larger RMS deviations observed in the binary simulation are driven by the structural relaxation of the CTD, while this remains relatively restricted in the ternary ones (i.e., in the presence of the substrate peptides). Time‐evolved RMSD variations for the p53 peptides show a stable profile for the methylated at Lys372 system (Model‐D; Fig. S2C). In contrast, more fluctuations are observed for the unmethylated peptide (Model‐B) and the p53 methylated at Lys370 (Model‐C). All the following analyses were restricted to the last 900 ns of the trajectories.

**Figure 2:**
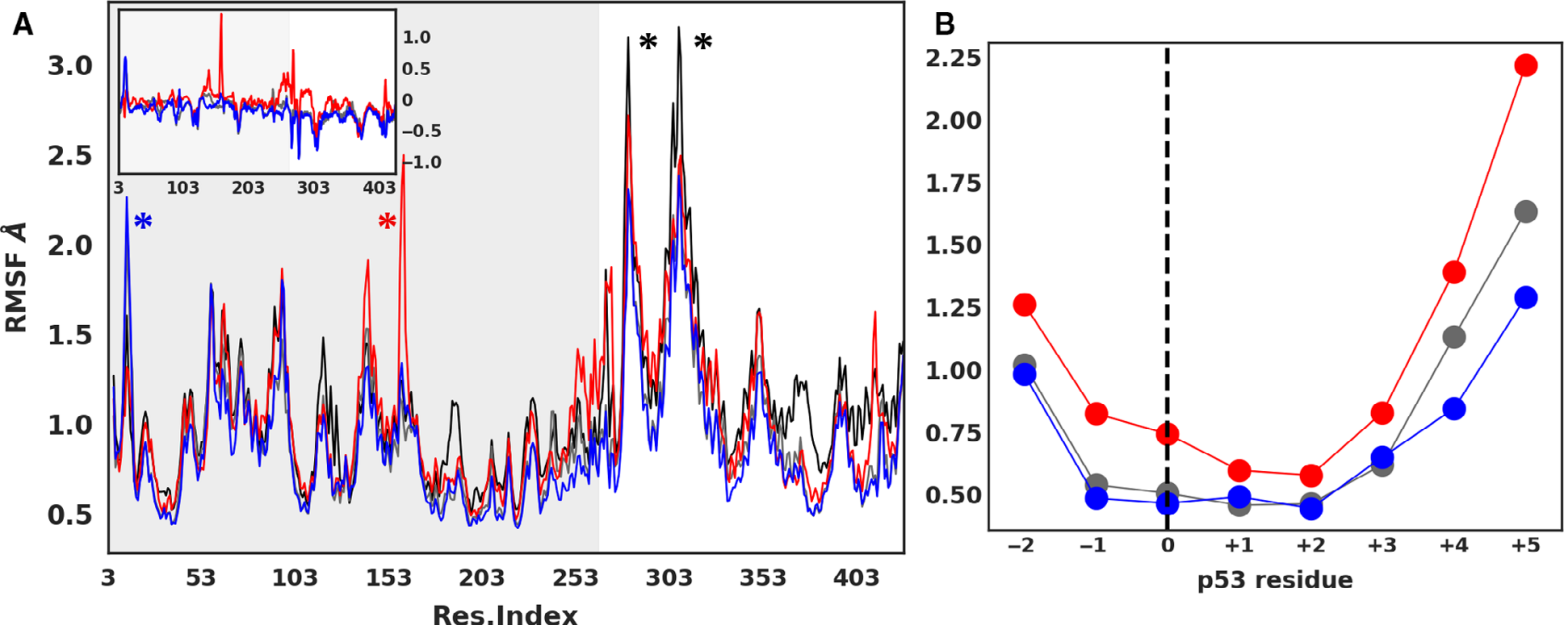
Residue flexibility in the simulated models. Per‐residue averaged backbone RMSF as a function of residue index. (A) RMSF for Smyd2 ignoring the terminal ones for models A–D (refer Fig. 1C) in black, gray, red, and blue, respectively. Peak regions are indicated with asterisks. Inset plot indicates the difference RMSF values for ternary systems with respect to the binary system. Shaded portion represents the N‐terminal domain. (B) RMSF for p53 residues in ternary systems (refer Fig. 1c for residue index definition).

To identify the flexible segments of Smyd2 protein and p53 peptides, we calculated the per‐residue averaged backbone root‐mean‐square fluctuation (RMSF) (Fig. 2A). In absence of the p53 peptides, the most mobile regions are located around the first antiparallel α‐helices of the C‐terminal domain (black asterisks). In the ternary systems, additional peaks are observed for models C (residue 157, red asterisk) and D (residue 10, blue asterisk), in the NTD. These residues belong to a small solvent‐exposed loop at the N‐terminal segment and MYND domain of Smyd2, afar sites from the peptide‐bound crevice.

The RMSF plots for the p53 peptides indicate a higher flexibility at the terminal ones (Fig. 2B). The flexibility is especially enhanced at the C‐terminal end of the peptide, which forms direct contacts with Smyd2′s CTD. The p53 peptide methylated at Lys370 exhibits higher mobility compared to the unmethylated or methylated at Lys372 one. The localized methylation effect on the residue flexibility was also examined by RMSF estimations at time windows of 30 ns. The obtained range of RMSF, depicted in Fig. S3, suggests that the methylation at Lys370 (position 0) or Lys372 (position +2) enhances the flexibility at the nearby sites (± 1 residues) in comparison with the unmethylated one. This effect is slightly higher for the Lys370 methylation.

### 3.2 C‐terminal domain exhibits different dynamical response to unmethylated and methylated p53 peptides

To characterize the global dynamics of Smyd2 across the different simulations, the dynamic residue cross correlation (DCC) and PCA were performed considering the backbone atoms. The DCC highlights the inter‐residue communications, even at long range. Positive and negative correlation (*C*_*ij*_) values indicate in‐phase and out‐of‐phase motions of a residue pair (*r*_*i*_ and *r*_*j*_). PCA permits to describe the complex protein motion in a subset of subspaces that largely accounts for most of the motional variance, as defined by the corresponding eigenvectors.

**Figure 3:**
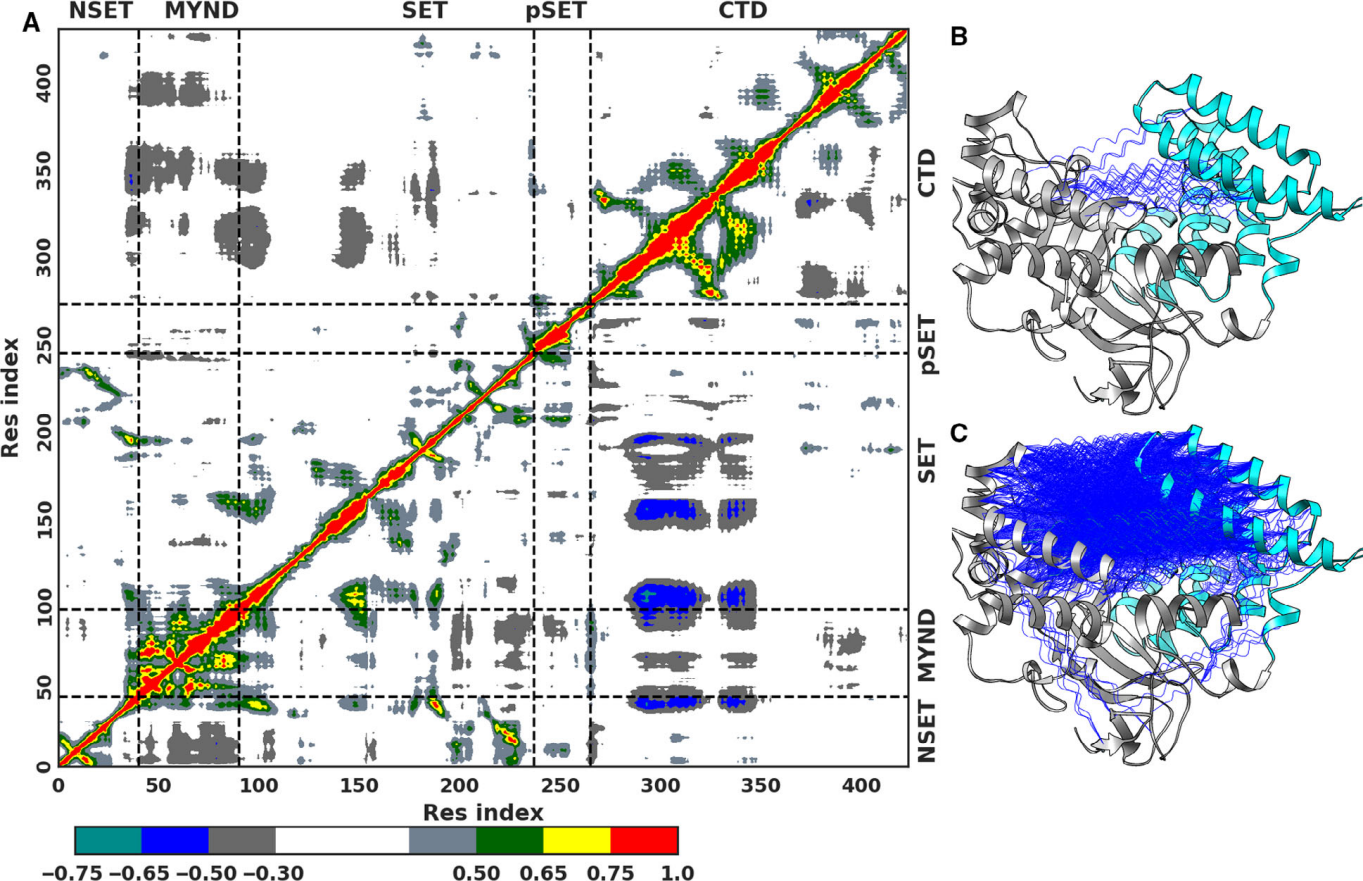
2D and 3D representations of dynamic cross correlation between protein residues. (A) Dynamic cross correlation matrix for Smyd2 residues for the ternary systems: Model‐B containing unmethylated‐p53 (upper triangle) and Model‐C with methylated‐p53 at Lys370 (lower triangle). (B) Structural mapping of the cross correlation values for Model‐B, where Cα pairs having correlations in range −0.7 < *C*_*ij* _< −0.5 are connected by blue springs. (C) Structural mapping of the cross‐correlation values for Model‐C.

The DCC matrix obtained for models B and C is shown in Fig. 3, and the matrix for models A and D is reported in Fig. S4. Inspection of the matrices reveals noticeable differences in interdomain communications between N‐ and C‐terminal domains. In Model‐C, the CTD exhibits strong anticorrelation movements against the NTD segments. This anticorrelation is highly reduced in Model‐B, containing the unmethylated peptide (Fig. 3A, compare blue regions in upper and lower triangles). Structural depiction of strong *C*_*ij*_ values highlights that the interdomain anticorrelations in Model‐C span across different segments of both the domains (Fig. 3B). In Model‐B, the anticorrelations are restricted between the first antiparallel α‐helices of the CTD and MYND segment of the NTD (Fig. S4). Methylation of Lys370, therefore, has as a consequence the establishment of new long‐range interactions, particularly between NTD and CTD.

The DCC matrix for Model‐A (Fig. S4A, upper triangle) shows strong anticorrelations between the two domains. This result is also consistent with the previously reported observation from sub‐μ‐second sampling for the model (Chandramouli and Chillemi, 2016). Smyd2 in Model‐D exhibits lower interdomain anticorrelations (Fig. S4A, lower triangle), similar to Model‐B (Fig. 3C, upper triangle). The structural depiction reveals again the presence of the corresponding correlations only between a fewer residues in the first α‐helix of the CTD and those in the MYND segment of the NTD. Methylation of Lys372 is not capable, therefore, to activate long‐range interactions, and in this respect, this system is similar to the unmethylated p53 peptide. At variance, several long‐range interactions are present in absence of the p53 peptide.

**Figure 4:**
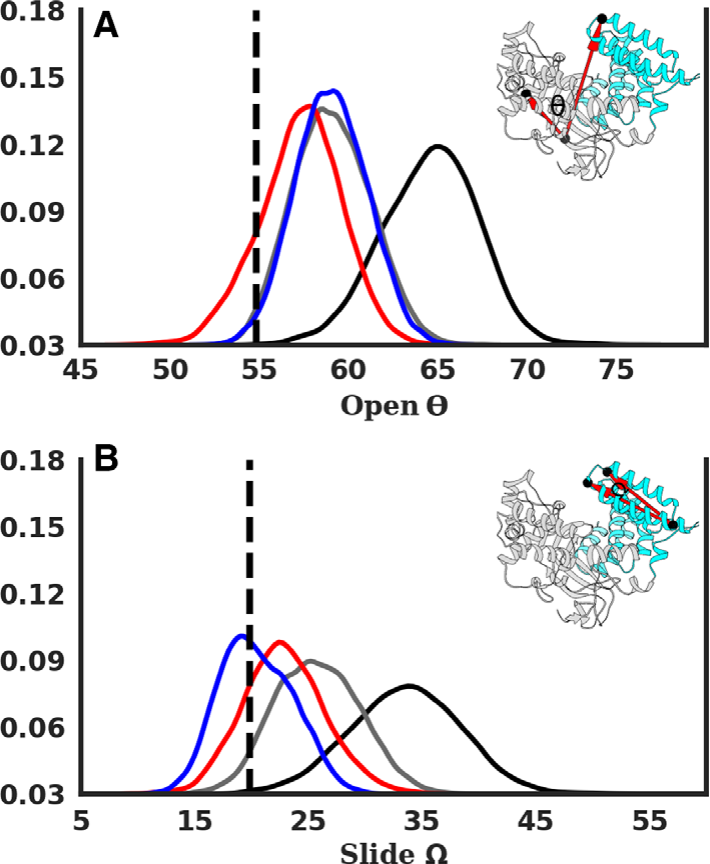
Geometric descriptors characterizing the hinge motion of the CTD for models A–D in black, gray, red, and blue, respectively (refer Fig. 2). (A) Definition of open angle along with its distribution over the last 900 ns. (B) Definition of slide angle and its corresponding distribution. The dashed line indicates the value for the X‐ray structure.

In order to further characterize the anticorrelated motions highlighted by the DCC analysis, we defined and analyzed two geometric descriptors, the open and slide angles, whose distribution is reported in Fig. 4. These highlight a sort of hingelike motion of the CTD with respect to the NTD counterpart. In the binary case (Model‐A), the distribution of both angles is well shifted from the X‐ray configuration and also above the ternary ones (models B–D). The mean values of the absolute difference in the open angles from the X‐ray structure for models A–D were 9.7 (± 2.7), 4.3 (± 2.1), 2.9 (± 1.8), and 4.2 (± 2.0) degree. Similarly, the mean values for the slide angles were 13.7 (± 4.9), 6.1 (± 3.5), 3.8 (± 2.9), and 2.7 (± 1.9) degree.

To compare the Smyd2′s dynamics across the simulations, PCA was performed on a concatenated trajectory that included snapshots from all the models. Projection of trajectory snapshots onto the subspace defined by first three eigenvectors that explains ~ 70% of motional variance is reported in Fig. S5. It is clear that the dynamical basin of Model‐A (binary system) is well separated from the ternary ones (Fig. S5A,B). Further, the larger deviation in mean projection along the corresponding eigenvectors confirms its higher flexibility compared to the ternary ones (Fig. S5C). Among the ternary ones, projections of Model‐D overlap yet span a restricted basin compared to the other ternary systems (models B and C).

### 3.3 p53 methylation at Lys372 has notable effects on the accessibility of Lys370 to the cofactor

**Figure 5:**
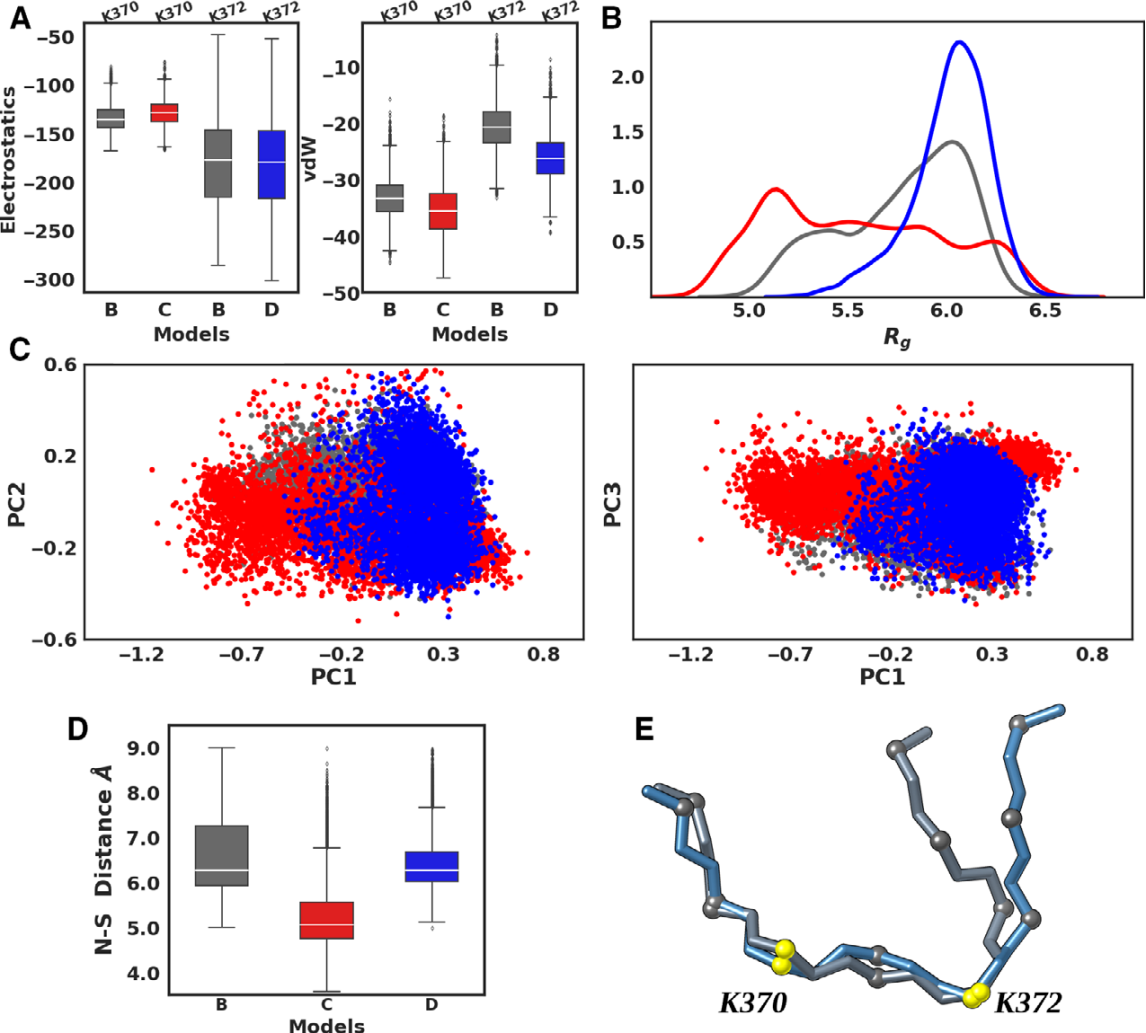
p53 interaction profile and conformational dynamics. (A) Boxplot representation of interaction energy (electrostatic and van der Waals terms in kcal·mol^−1^) between the methylation site (i.e., Lys370/Lys372 and ± 1 adjacent residues) and Smyd2 for models B–D (refer Fig. 1c) in gray, red, and blue, respectively. (B) Distribution of the gyration radius of p53 peptide during the last 900 ns. (C) Projection of p53 structures along the eigen subspace defined by principal vectors 1–2 and 1–3, respectively. (D) Distance between Lys370 amino group nitrogen and the sulfur atom of AdoMet or AdoHcy. (E) Superimposition of representative conformers obtained from clustering.

We characterized the dynamical features of bound p53 peptides in the ternary complexes by examining the interaction profile, geometric, and structural parameters (Fig. 5). Interaction energy between the p53 methylation sites (Lys370/Lys372 ± 1 residue) and Smyd2 residues within 15 Å was estimated via electrostatic and vdW terms and represented as boxplots (Fig. 5A). Methylation at Lys370 or Lys372 does not have great influence on the electrostatic interactions (compare gray vs red bars for Lys370 and gray vs blue bars for Lys372). On the contrary, the vdW interactions are increased as indicated by the reduction by ~ 3.2 and 6 kcal·mol^−1^ of the median values (white line in Fig. 5A) in models C and D, respectively. The Q1–Q3 quartile range distribution represented in the boxplot, however, shows that the two distributions are significantly different only in the case of methylation at Lys372 (model‐D vs the unmethylated model‐B). Interaction energies between the complete p53 peptide and Smyd2 show a similar trend: no distinct difference in electrostatic interactions except an increase in vdW interactions as we go from models B to D (Fig. S6A). Also, in this case, the boxplot distributions for models D and B are significantly different. Distribution of the gyration radius reveals that p53 methylated at Lys372 maintains a relatively compact structure as compared to the unmethylated or methylated p53 at Lys370. This is also apparent from the time‐evolved variations in gyration radius (Fig. S6B). Further, the PCA and corresponding projections along the principal eigenvectors (Fig. 5C) reveal a restricted conformational basin for Lys372‐methylated p53, which correlates with other structural characteristics mentioned above.

The accessibility of Lys370, the natural target site for Smyd2‐mediated p53 methylation, to the cofactor was assessed by calculating the distance between the amino group of Lys370 and the sulfur atom of AdoMet/AdoHcy that acts as the methyl donor (boxplot representation in Fig. 5D). Lys370 in Model‐C is more accessible than when the target peptide is unmethylated (Model‐B). Further, methylation at Lys372 also influences Lys370 accessibility to the sulfur as demonstrated by the increased distance by ~ 1.4 Å, even though the difference between the distance distribution in models D and B is not significant. These observations correlate with the increased vdW interactions between the methylated lysines and protein residues (*vida supra*).

Biochemical characterization of Smyd2 activity via steady‐state kinetic and product inhibition experiments has established that Smyd2 operates through a rapid equilibrium random Bi Bi mechanism (Wu *et al*., 2011). The lysine methylation involves a direct nucleophilic attack of the methyl group leaving the AdoMet by the target amine without the formation of methyl‐protein intermediate (Wu *et al*., 2011). Hence, a proper insertion of the target lysine (Lys370 in this case) to access of the methyl group in the deep crevice is indispensable for methylation. Further, Lys372 methylation is shown to partially block the Smyd2‐p53 interaction (Huang *et al*., 2006). Taking together, the current data also highlight the molecular basis for the inhibition of Lys370 methylation by monomethylated Lys372.

To extract representative conformers that can better explain the conformational rearrangements, density‐based clustering analysis was performed based on the peptide structural properties (Fig. S7). The representative conformers, extracted from the high dense regions, are reported in Fig. 5E. The conformers are well superimposed between positions −1 to +2 (Leu369–Lys372) except at the terminals with high degree of difference at the C‐terminal end that engages in interactions with the Smyd2 CTD. This result agrees with the larger flexibility observed for this region (*vide supra*). It is also worth to note that the U‐shaped conformation of the peptide is well maintained as observed in the X‐ray structure.

The comparison of Smyd2 structures in complex with ERα and p53 peptides showed that both peptides adopt similar U‐shaped conformation, the respective target lysines being similarly orientated toward the cofactor (Jiang *et al*., 2014). However, structural difference was observed at residues spanning beyond +3 positions from the target lysine, interacting with distinct residues in the CTD. In line, representative conformers, reported here, show notable difference toward the C‐terminal ends. This explains the role of the CTD in substrate stabilization via characteristics interactions.

## 4 Conclusions

The p53 is the most frequently mutated gene in cancer, reaching a median frequency of 50% mutation rate in all cancers. This results in gain‐of‐function deregulation of the DNA damage response (Vaughan *et al*., 2017; Zache *et al*., 2017), fostering tumor progression (Bartek *et al*., 2017; Fang *et al*., 2018), for example, by deregulating its apoptotic targets (Goiran *et al*., 2018; Sharma *et al*., 2018), metabolic enzymes (Moon *et al*., 2019; Sorrentino *et al*., 2018; Xu *et al*., 2018), or the AMPK pathway (Houde *et al*., 2017). Understanding these mechanisms is essential to explore innovative therapeutic venues (Hasna *et al*., 2018; Malgerud *et al*., 2017; Siebring‐van Olst *et al*., 2017). At the molecular level, p53 physically binds several targets, such as, for example, MDM2 and MDMX (Arena *et al*., 2018; Soares *et al*., 2017) or indeed Smyd2 (Wang *et al*., 2011). Based on the structural insights on p53 (Joerger and Fersht, 2008, 2016), we have therefore investigated the interaction between p53 and Smyd2.

Thanks to the availability of a supercomputer, we have performed an unusual long MD analysis, up to four μ‐second total simulated time. The data shown above sustain the conclusion that the C‐terminal domain of Smyd2 (a) has a degree of conformational freedom that is restricted by the physical interaction with the p53 peptide substrate, and (b) shows distinct dynamic properties when interacting with unmethylated and methylated p53 peptides. In turn, (c) the p53 peptide is conformationally confined by Lys372 methylation, which also noticeably affects the Lys370 accessibility to the cofactor. These MD results further expand the knowledge of the p53 biology at the molecular level.

## Conflict of interest

The authors declare no conflict of interest.

## Author contributions

BC and GC conceived and performed the MD experiments; all authors wrote the paper.

## Supporting information


**Fig. S1.** Depiction of the ternary complex and cofactor binding cavity.
**Fig. S2.** Time evolved RMSD variations and distributions.
**Fig. S3.** Minimum and maximum fluctuations of p53 peptide residues.
**Fig. S4.** Dynamic cross correlation matrix for Models A and D.
**Fig. S5.** PCA projections for Smyd2 along principal eigen vectors.
**Fig. S6.** Interaction energy and gyration radius of p53 peptide for models‐B–D.
**Fig. S7.** Density based clustering of p53 peptide conformers.
