# Supplementary material for: Smyd2 conformational changes in response to p53 binding: role of the C‐terminal domain
Source: Mol Oncol. 2019 May 21;13(6):1450–61. doi: 10.1002/1878-0261.12502 (PMC6547616; doi:10.1002/1878-0261.12502)
Supplement: Supplementary file 1 — Fig. S1. Depiction of the ternary complex and cofactor binding cavity. Fig. S2. Time evolved RMSD variations and distributions. Fig. S3. Minimum and maximum fluctuations of p53 peptide residues. Fig. S4. Dynamic cross correlation matrix for Models A and D. Fig. S5. PCA projections for Smyd2 along principal eigen vectors. Fig. S6. Interaction energy and gyration radius of p53 peptide for models‐B–D. Fig. S7. Density based clustering of p53 peptide conformers. [file MOL2-13-1450-s001.pdf]

SUPPORTING INFORMATION

Smyd2 conformational changes in response to p53 binding: role of the C-terminal domain

Balasubramanian Chandramouli, Gerry Melino, Giovanni Chillemi

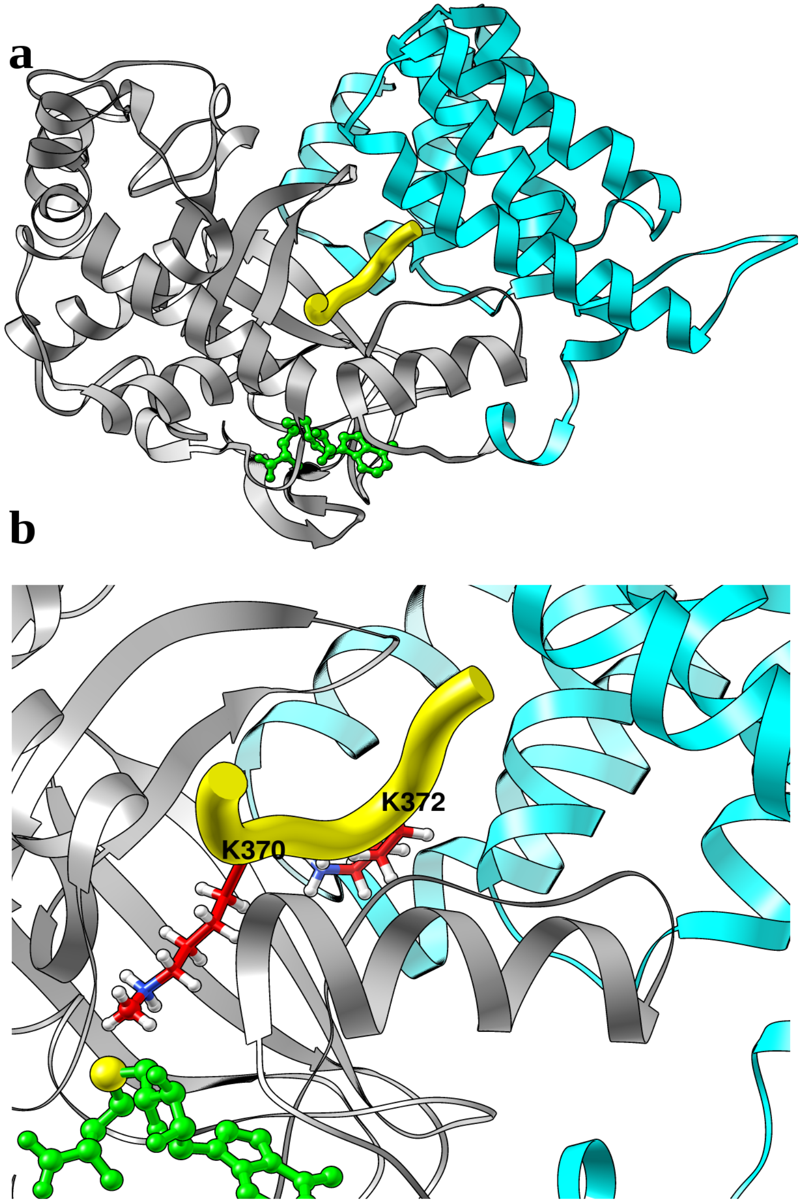

Ternary complex and cofactor binding cavity. a) Top view of ternary Smyd2-AdoHcy-p53 peptide complex depicting the U-shaped conformation of p53 peptide in the cradle between the Smyd2 N and C-terminal domains. b) Magnified view of the insertion of target Lys370 into the cofactor binding cavity. The nearby Lys372 site is shown for clarity. AdoHcy (green) whose sulfur atom that donate the methyl group (yellow sphere) is shown in ball-stick representation.

Fig. S1

Chandramouli et al.

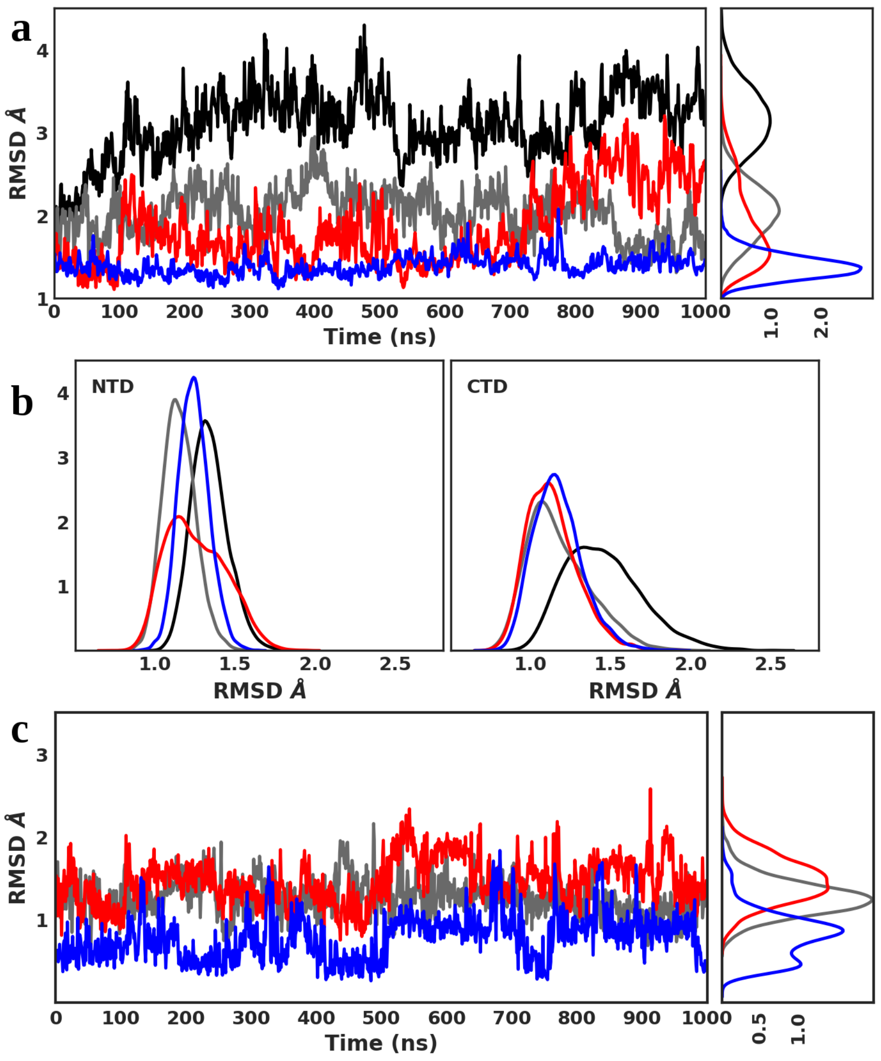

Time evolved variations in the structural deviation. a) RMSD as a function of simulation time for Smyd2 and corresponding distribution over the last 900 ns. b) Distribution of RMSD values (over last 900 ns) for Smyd2, estimated separately for the N and C-terminal domains. c) RMSD of p53 peptide in the ternary systems.

Fig. S2

Chandramouli et al.

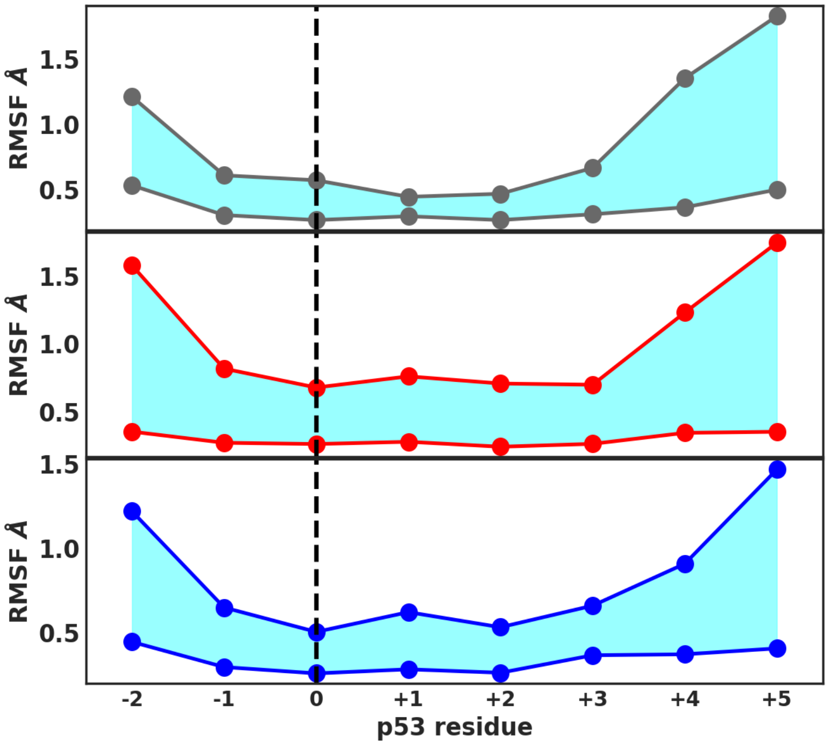

Minimum and maximum fluctuation as a function of residue index. Flexibility range of p53 residues in ternary complex simulations obtained by estimating the RMSF values in windows of 30 ns. Dashed line indicates Lys370.

Fig. S3

Chandramouli et al.

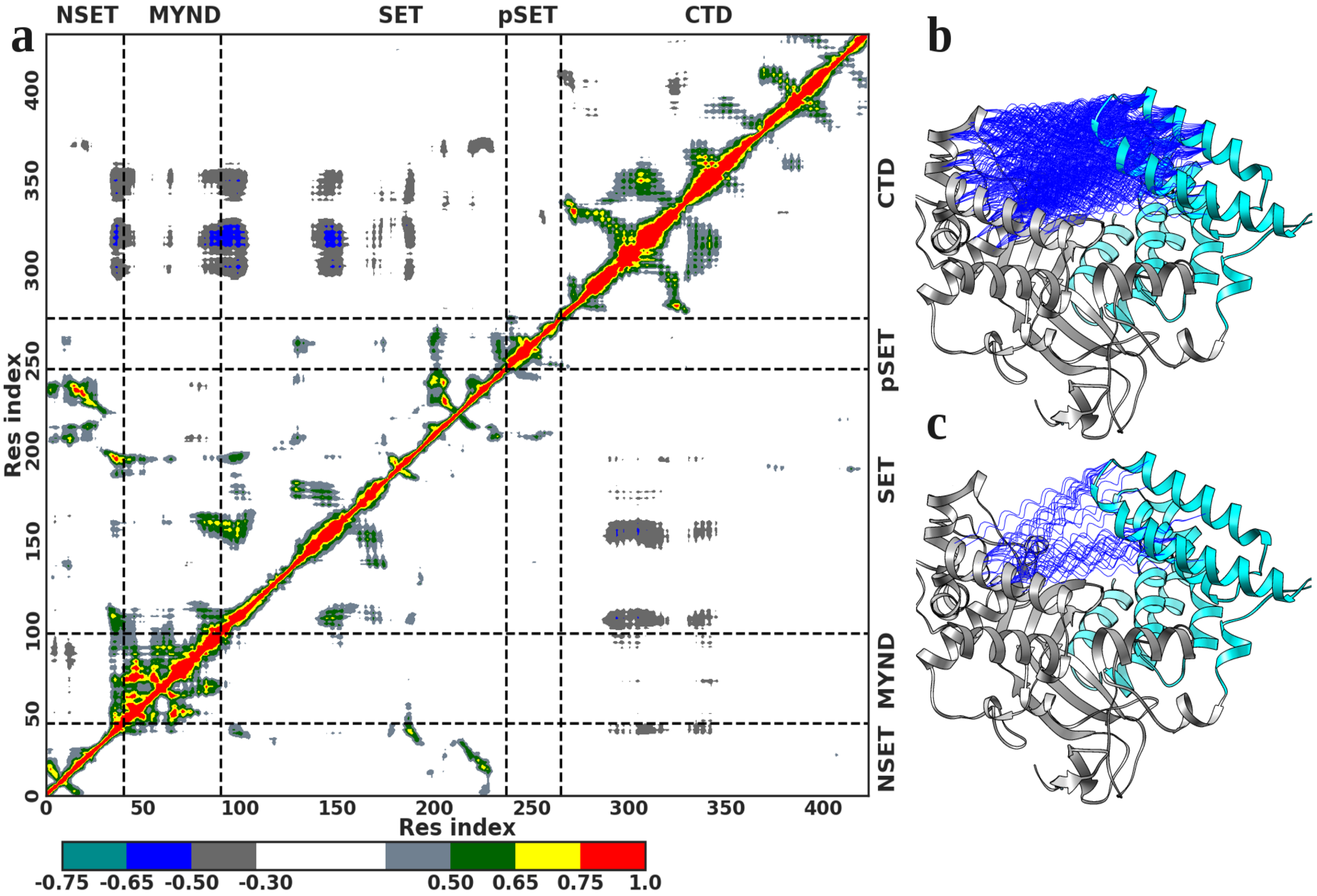

Dynamic cross correlation matrix. a) Binary (Model-A, upper triangle) vs ternary with methylated-p53 at Lys372 (Model-D, lower triangle) systems. b) Structural mapping of cross correlation values for Model-A, where Cα pairs having correlation in range -0.7 < Cij < -0.5 are connected by blue springs. c) Structural mapping of cross correlation values for Model-D.

Fig. S4

Chandramouli et al.

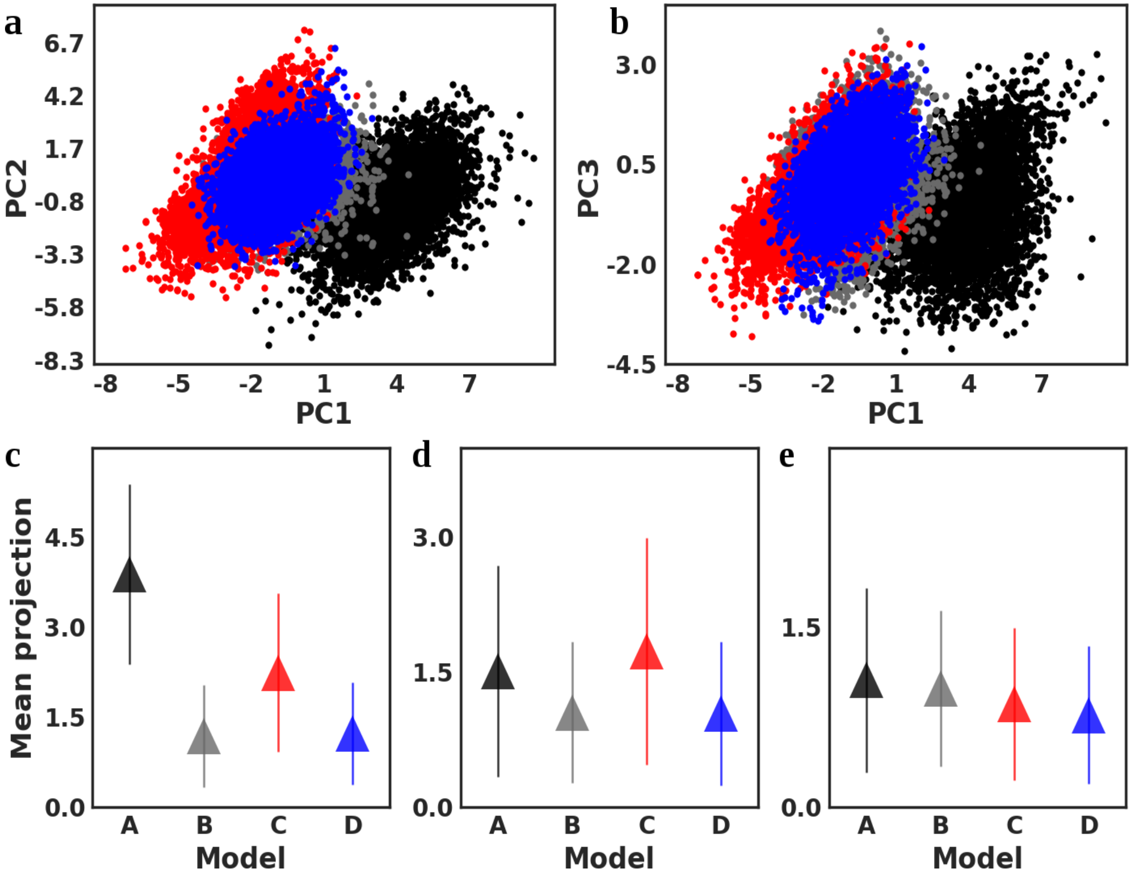

PCA projections along principal eigen vectors. a) Projection of frames along the eigen subspace defined by principal vectors 1,2. b) Projection of frames along the subspace defined by vectors 1,3. c) Mean projection along with std. Deviation (error bar) along principal vector 1. d-e) Same as c) along principal vectors 2-3.

Fig. S5

Chandramouli et al.

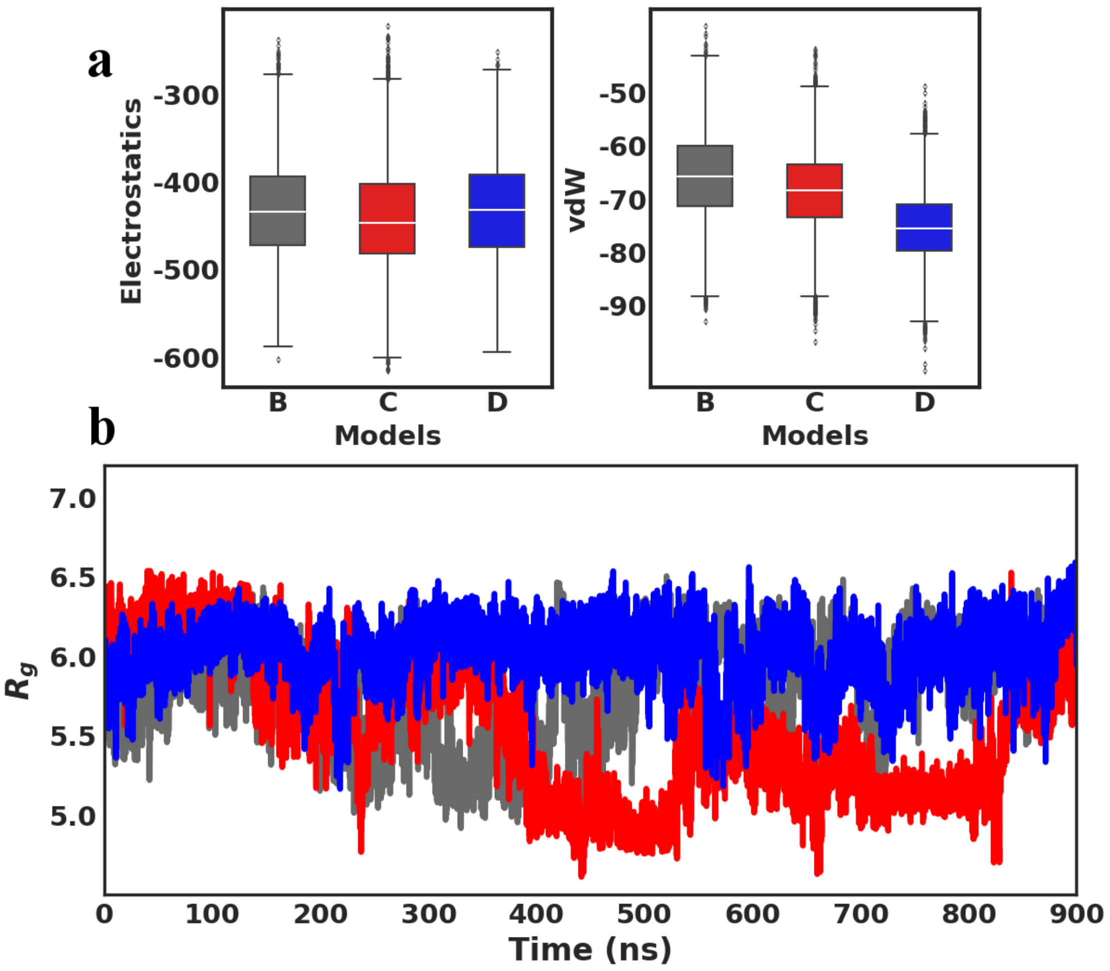

Interaction energy of the p53 peptide and gyration radius for models B-D (refer Fig. 1c) in gray, red and blue, respectively. a) Interaction energy (electrostatic and van der Waals terms in kcal.mol^{-1}) between p53 peptide and Smyd2 residues within 25 Å from the peptide reported as box-plot. b) Time evolved variation of gyration radius of p53 peptide.

Fig. S6

Chandramouli et al.

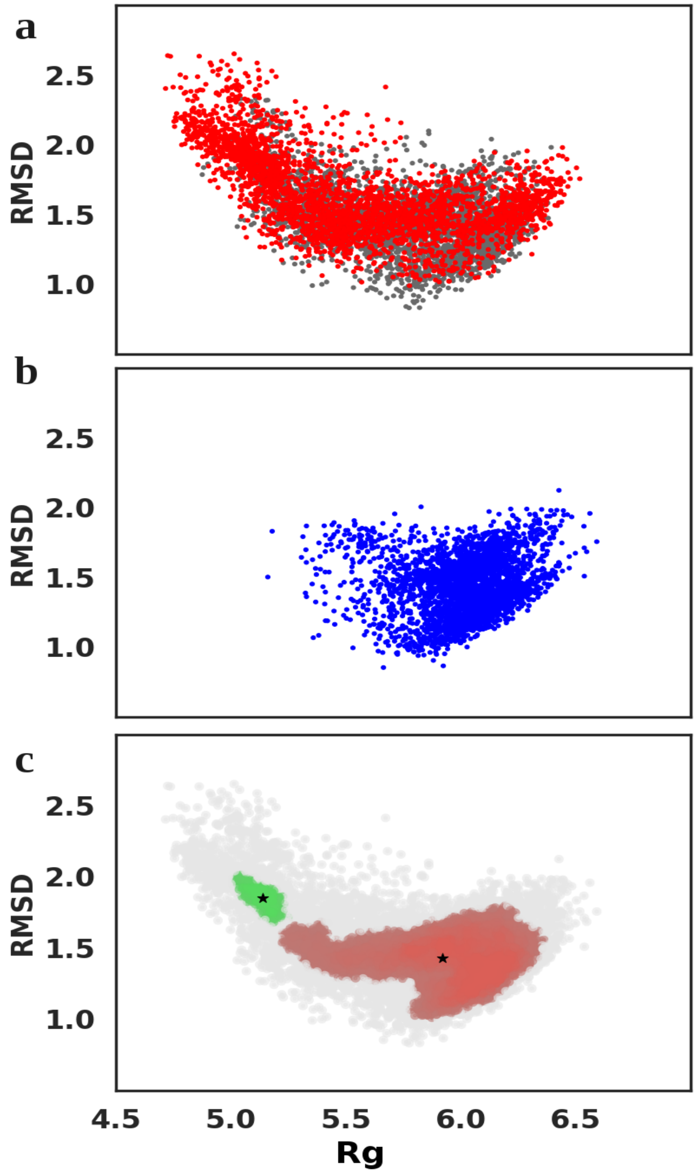

Density based clustering of p53 peptide conformers. a-c) Plot of RMSD vs Radius of gyration for the p53 peptide conformers belonging to Model-B and C (a), Model-D (b), and corresponding clustering based on density (c). Points populating the two dense clusters are shown in red and green, while the points spanning the less dense regions are shown in gray. Asterisks indicate representative conformers.

Fig. S7

Chandramouli et al.
